# Gastrodin ameliorates cognitive dysfunction in diabetes by inhibiting PAK2 phosphorylation

**DOI:** 10.18632/aging.204970

**Published:** 2023-08-22

**Authors:** Zhi-Hao Mu, Zhi-Min Zhao, Su-Su Yang, Lei Zhou, Yi-Dan Liu, Zhong-Yi Qian, Xin-Jie Liu, Peng-Chao Zhao, Ren-Bo Tang, Jia-Yin Li, Jing-Yao Zeng, Zhi-Hong Yang, Yong-Hua Ruan, Ying Zhang, Yue-Qin Zeng, Ying-Ying Zou

**Affiliations:** 1 Department of Pathology and Pathophysiology, Faculty of Basic Medical Sciences, Kunming Medical University, Kunming, China; 2 Department of Pathology and Pathophysiology, Baoshan College of Traditional Chinese Medicine, Baoshan, China; 3 College of Clinical Oncology, Kunming Medical University, Kunming, China; 4 Academy of Biomedical Engineering, Kunming Medical University, Kunming, China; 5 Institute of Drug Discovery and Development, Kunming Pharmaceutical Corporation, Kunming, China; 6 Department of Morphological Laboratory, Faculty of Basic Medical Sciences, Kunming Medical University, Kunming, China; 7 The Second Faculty of Clinical Medicine, Kunming Medical University, Kunming, China; 8 The First Faculty of Clinical Medicine, Kunming Medical University, Kunming, China; 9 School of Nursing, Kunming Medical University, Kunming, China; 10 Yunnan Key Laboratory of Stem Cell and Regenerative Medicine, Kunming Medical University, Kunming, China

**Keywords:** cognitive dysfunction, diabetes, Gastrodin, hippocampus, p21 activated kinase 2

## Abstract

Diabetes is associated with higher prevalence of cognitive dysfunction, while the underlying mechanism is still elusive. In this study, we aim to explore the potential mechanism of diabetes-induced cognitive dysfunction and assess the therapeutic effects of Gastrodin on cognitive dysfunction. Diabetes was induced by a single injection of streptozotocin. The Morris Water Maze Test was employed to assess the functions of spatial learning and memory. Transcriptome was used to identify the potential factors involved. Western blot and immunofluorescence were applied to detect the protein expression. Our results have shown that spatial learning was impaired in diabetic rats, coupled with damaged hippocampal pyramidal neurons. Gastrodin intervention ameliorated the spatial learning impairments and neuronal damages. Transcriptomics analysis identified differential expression genes critical for diabetes-induced hippocampal damage and Gastrodin treatment, which were further confirmed by qPCR and western blot. Moreover, p21 activated kinase 2 (PAK2) was found to be important for diabetes-induced hippocampal injury and its inhibitor could promote the survival of primary hippocampal neurons. It suggested that PAK2 pathway may be involved in cognitive dysfunction in diabetes and could be a therapeutic target for Gastrodin intervention.

## INTRODUCTION

Cognitive dysfunction has been recognized as a common complication and comorbidity of both type 1 and type 2 diabetes [1]. Up to 20% of type 2 diabetic patients older than 60 years could have dementia [2]. In addition to its well-known impact on peripheral tissues, diabetes has been linked to cognitive dysfunction, contributing to increased susceptibility to neurodegenerative disorders such as Alzheimer’s disease and vascular dementia. Cognitive dysfunction in diabetes (CID) includes dysfunction in memory, executive function, language, and spatial ability [3].

The molecular mechanisms underlying the relationship between diabetes and cognitive impairment have been the subject of extensive research. Several key processes have been identified that elucidate the intricate interplay between diabetes and cognitive dysfunction. One of the primary processes involved is chronic hyperglycemia-induced oxidative stress. Oxidative stress disrupts normal cellular functions, triggers inflammation, and promotes the formation of advanced glycation end products (AGEs) [4]. Accumulation of AGEs further exacerbates oxidative stress and activates inflammatory pathways, leading to neuronal dysfunction and synaptic impairment [5].

Another contributing factor is insulin resistance and impaired insulin signaling in the brain. Insulin plays a crucial role in regulating glucose metabolism and neuronal functions. In diabetes, reduced insulin availability or impaired insulin receptor signaling compromises glucose utilization and impairs synaptic plasticity and cognitive processes [6]. Furthermore, chronic low-grade inflammation, characterized by increased levels of pro-inflammatory cytokines, is a common feature of diabetes and has been implicated in cognitive decline. Inflammatory mediators disrupt neuronal function and contribute to neuroinflammation, synaptic loss, and impaired cognitive performance [7].

The hippocampus plays a key role in cognitive functions, which is particularly vulnerable to metabolic diseases and age-related neurodegeneration. During the progression of Alzheimer’s disease, the hippocampal CA1 pyramidal neurons are selectively attacked [8]. Patients with diabetes exhibited hippocampal insulin resistance and neuronal loss in the hippocampal CA1 region, which are potential mediators of CID [9]. The decline of hippocampal neuronal function is closely related to cognitive dysfunction caused by degenerative diseases [10].

In recent years, natural compounds with potential neuroprotective effects have gained attention in mitigating diabetes-associated cognitive dysfunction. *Gastrodia elata* is a traditional Chinese herbal medicine, which has been used for the treatment of headache, dizziness, epilepsy, stroke, and amnesia for a long time [11]. Gastrodin, the main active ingredient of *Gastrodia elata*, also known as 4-hydroxybenzyl alcohol-4-O-β-D-glucopyranoside (PubChem CID: 115027), is a phenolic glycoside, whose therapeutical effects on central nervous system diseases has been frequently reported recently [12]. It works through regulating neurotransmitters, such as GluR2 and NR2A [13], restoring vascular function [14], as well as having anti-oxidation and anti-inflammatory effects [15]. Several neurotransmitters influenced by *Gastrodia elata* include acetylcholine, dopamine, and serotonin [16]. But the effect of Gastrodin on spatial learning and its possible target has remained elusive.

Considering the above, we hypothesized that Gastrodin could improve spatial learning in diabetic rats through protecting hippocampal neurons. This study sought to assess the effect of Gastrodin on CID through transcriptome analysis and confirm the role of candidate factors in the pathogenesis of CID. Therefore, RNA-seq was used to reveal the expression changes of significant differential genes between the hippocampus of diabetic rats and normal controls.

## RESULTS

### Gastrodin intervention improved cognitive function and ameliorated pathological changes in diabetic rats

**Figure 1:**
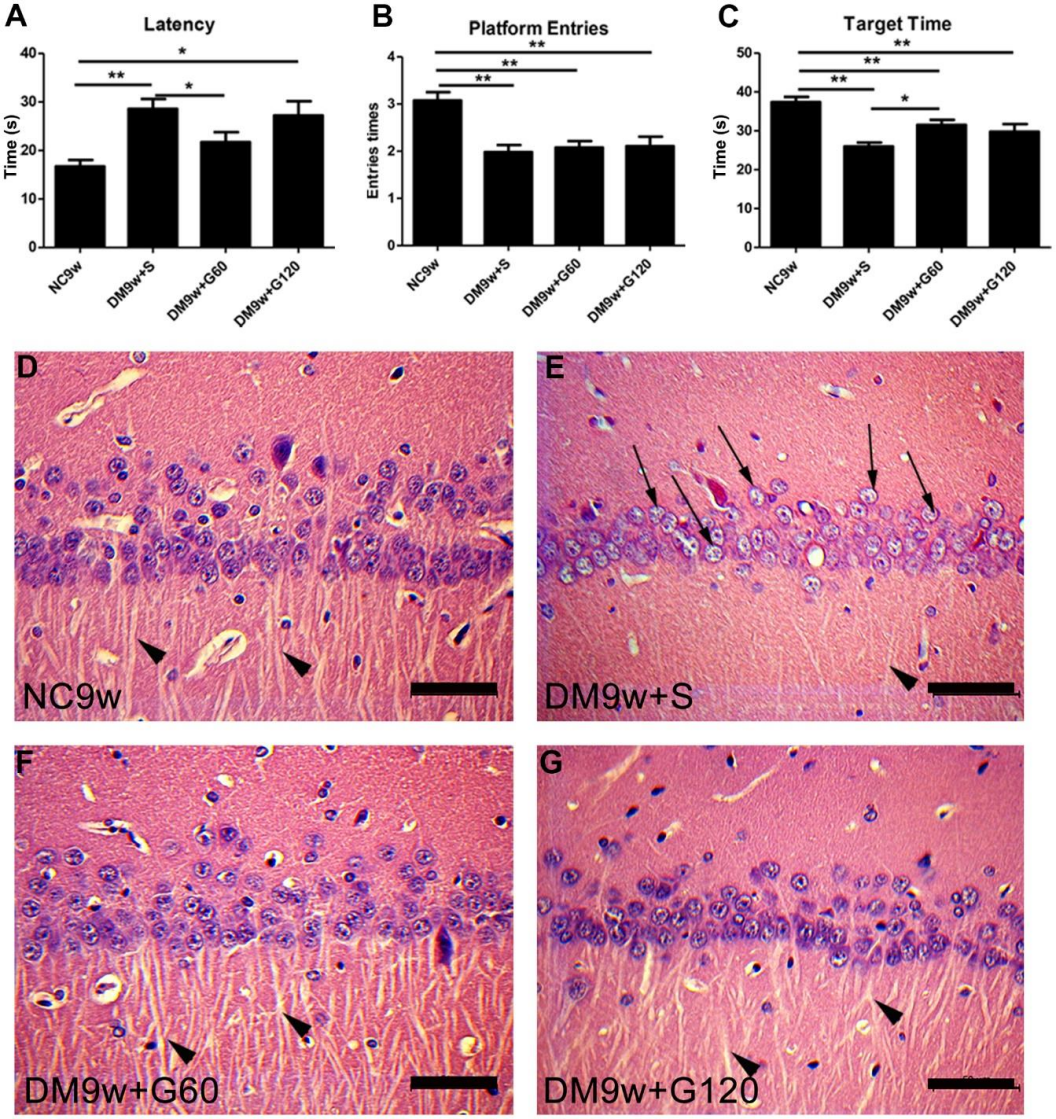
**Gastrodin intervention ameliorated diabetes-induced cognitive dysfunction and pathological changes in the CA1 area of the hippocampus.** Escape latency (**A**) number of platform entries (**B**) and time spent in target quadrant (**C**) of rats from the NC9w, DM9w+S, DM9w+G60 and DM9w+G120 groups in the probe trial. (**D**–**G**) H&E staining of CA1 area of hippocampus in the NC9w, DM9w+S, DM9w+G60 and DM9w+G120 groups. Black arrows indicated the nucleus of the damaged neuron and arrowheads indicated the synapse. Bar = 50 μm. *p < 0.05, **p < 0.01.

To evaluate the effects of Gastrodin intervention on cognitive function, Morris water maze test was performed. Diabetic rats exhibited significantly higher escape latency than the controls (p < 0.01), which was significantly decreased after 60 mg/kg of Gastrodin intervention (Figure 1A). In the space exploration stage, the number of platform crossing and the target quadrant dwelling time were significantly decreased in the DM9w+S group than that of the NC9w group (p < 0.01). 60 mg/kg of Gastrodin intervention significantly increased the target quadrant dwelling time of diabetic rats, while it had no effect on the number of platform crossings (Figure 1B, 1C). Interestingly, 120 mg/kg of Gastrodin intervention showed no therapeutic effect on the performance of Morris water maze test.

H&E staining showed that neurons in the CA1 pyramidal region of the hippocampus had an intact structure and clear nuclei in the NC9w group. In addition, the axon of the radiatum layer was extended and arranged neatly. In contrast, the DM9w+S group displayed a notable reduction in the number of pyramidal neurons in the hippocampal CA1 region. Additionally, the cytoplasm was diminished, the nuclei showed signs of edema, and the axon extension in the radiatum layer was both reduced and disorganized. Comparatively, both the DM9w+G60 and DM9w+G120 groups exhibited a higher number of pyramidal neurons in the hippocampal CA1 area than the DM9w+S group. Moreover, neurons in both groups displayed abundant cytoplasm, clear nuclei, and neatly arranged axons in the radiatum layer (Figure 1D–1G).

### Screening possible pathways underlying diabetes-induced cognitive dysfunction and Gastrodin intervention by RNA-seq analysis

**Figure 2:**
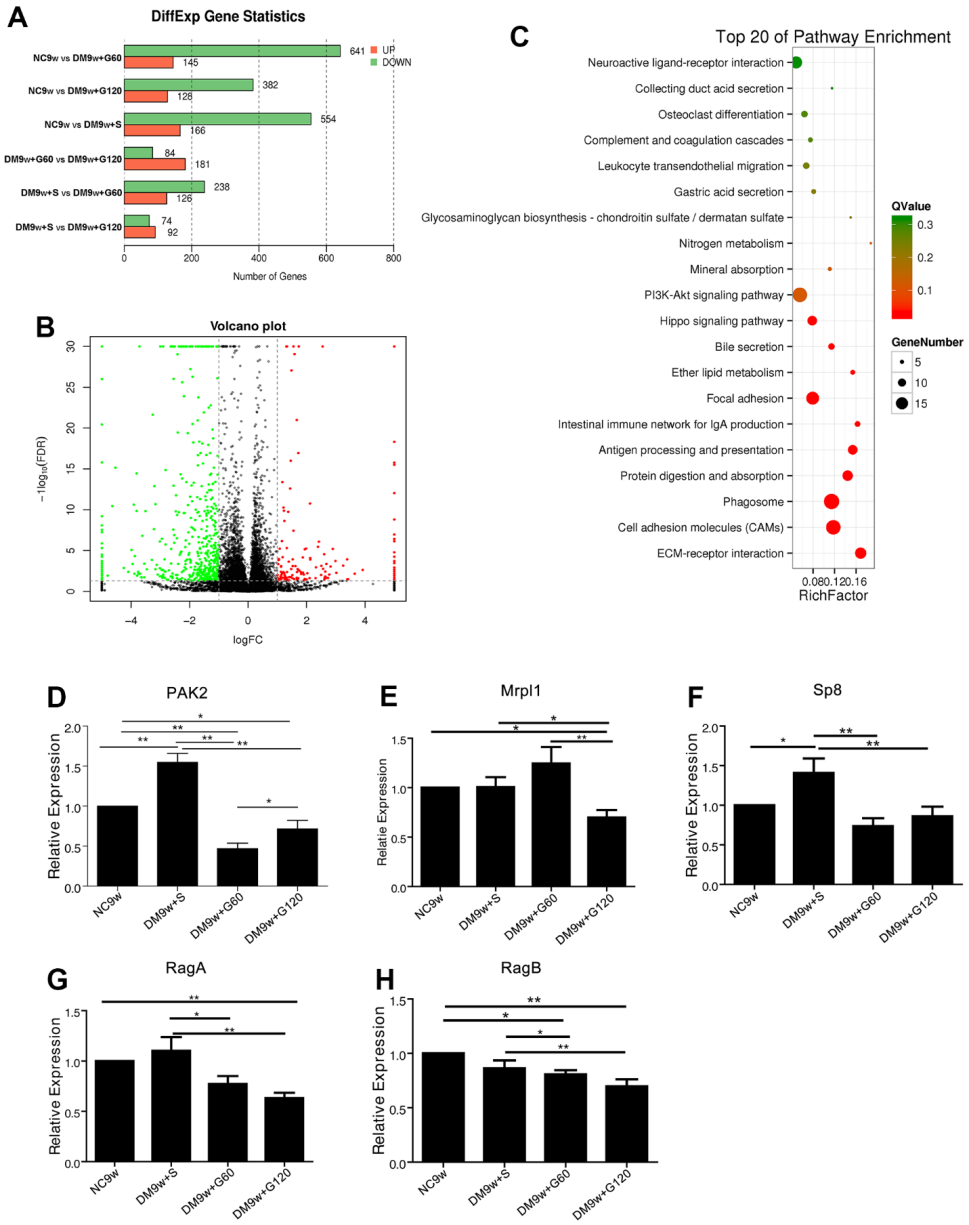
**The transcriptomic profile of Gastrodin intervention on the hippocampus of diabetic rats and further confirmation.** (**A**) Differentially expressed genes among NC9w, DM9w+S, DM9w+G60 and DM9w+G120 group. (**B**) Volcano plot of all genes based on their log2 fold-change and adjusted P-values between NC9w and DM9w+S group. Differentially expressed genes were classified at an adjusted P-value of < 0.05. (**C**) The KEGG pathways enriched between NC9w and DM9w+S group. The rich factor refers to the ratio of the number of DEGs to the number of total annotated genes in a certain pathway. (**D**–**H**) qPCR analysis of the mRNA expression of PAK2 (**D**) Mrpl1 (**E**) Sp8 (**F**) RagA (**G**) and RagB (**H**) in the hippocampus of the NC9w, DM9w+S, DM9w+G60 and DM9w+G120 groups. *p < 0.05 and **p < 0.01.

The differentially expressed genes (DEGs) were screened by FDR and log2FC under the conditions of FDR<0.05 and |log2FC| >0.1. Compared with the NC9w group, DM9w+S group had 166 upregulations of DEGs and 554 downregulations; DM9w+G60 group had 145 u-regulations and 641 downregulations; DM9w+G120 group had 128 upregulations and 382 downs on DEGs. Compared with the DM9w+S group, there were 126 up-regulation and 238 down-regulation of DEGs in the DM9w+G60 group; 92 up-regulation and 74 down-regulation in the DM9w+G120 group. Compared with the DM9w+G60 group, there were 181 up-regulations and 84 down-regulations in the DM9w+G120 group (Figure 2A). The DEGs between NC9w and DM9w+S were presented by volcano plot (Figure 2B).

The properties of DEGs were described according to the internationally standardized gene function classification system Gene Ontology (GO). The molecular function, cellular component and biological progress of the genes were respectively described. GO analysis showed that the main modules of these DEGs are cellular process, transmitter transport and extracellular component changes. Pathway enrichment analysis with KEGG Pathway was applied to find significant enrichment pathways in sub-differential expression compared with the entire genetic background. The first 20 significantly enriched pathways between different groups were found, which included cell adhesion molecule pathway, phagocytic pathway, extracellular matrix receptor interaction pathway, PI3K-AKT signaling pathway, focal adhesion pathway, etc. (Figure 2C).

Next, DEGs were further screened with two criteria: fold change ≥ 5 and its expression pattern should show the same pattern as the behavioral changes (Table 1). If it’s upregulated in DM9w group, it must be downregulated in both DM9w+G60 and DM9w+G120 group. Finally, according to literature, PAK2, Mrpl1, RagA/B and Sp8 were selected from the key genes for further confirmation. qPCR test showed that the expression of Mrp11, RagA and RagB in DM9w+S group was not significantly higher than that of the NC9w group. By contrast, the expression of PAK2 and Sp8 was significantly increased after diabetes induction. In addition, their expressions in the DM9w+G60 and DM9w+G120 groups were significantly lower than these of the DM9w+S group, which were consistent with transcriptome changes (Figure 2D–2H). Because diabetic rats exhibited impaired learning ability and Gastrodin had a therapeutic effect, we reviewed the reports on cognition and found that PI3K-AKT signaling pathway was associated with insulin-mediated cognitive function [17, 18]. Moreover, PAK2 was closely related with PI3K-AKT insulin signaling, which downwardly resulted in reduced glucose uptake under pathological conditions through glucose transduction receptors GLUT4. It would lead to the damage of insulin-dependent neurons and compromised cognitive function. Therefore, PAK2 and PI3K/AKT/GLUT4 signaling pathway was selected for further investigation.

**Table 1:** Key genes screened by fold change and behavioral outcome.

**Gene ID**	**Symbol**	**Pathway**	**GO component**	**GO function**	**GO process**
ENSRNOG00000002070	Mrpl1	ko03010//Ribosome	cytoplasmic part	nucleic acid binding	peptide metabolic process
ENSRNOG00000009331	Hck	ko04062//Chemokine signaling pathway	cellular component	transferase activity	organelle organization
ENSRNOG00000010597	Slc5a7	ko04725//Cholinergic synapse	intracellular organelle	transporter activity	transmembrane transport
ENSRNOG00000012686	Pomc	ko04916//Melanogenesis	extracellular space	molecular function	regulation of biological process
ENSRNOG00000018827	Htr7	ko04014//Ras signaling pathway	intracellular membrane-bounded organelle	signal transducer activity	single-organism cellular process
ENSRNOG00000047864	RT1-DMa	ko04612//Antigen processing and presentation	plasma membrane	—	biological process
ENSRNOG00000048597	Pak2	ko04010//MAPK signaling pathway	membrane-bounded vesicle	catalytic activity	cellular protein metabolic process
ENSRNOG00000003160	LOC100909655	mTOR signaling pathway	endosome	molecular function	response to stimulus
ENSRNOG00000004372	Cbln4	—	cellular component	molecular function	biological process
ENSRNOG00000005387	Rbm3	—	intracellular membrane-bounded organelle	heterocyclic compound binding	—
ENSRNOG00000005943	Sp8	—	—	binding	single-multicellular organism process
ENSRNOG00000011459	Rhbdf2	—	endoplasmic reticulum	—	regulation of biological process
ENSRNOG00000019351	LOC100911881	—	cellular component	catalytic activity	carbohydrate metabolic process
ENSRNOG00000031743	Gbp2	—	cell	molecular function	biological process
ENSRNOG00000047651	ProSAP1	—	—	—	—
ENSRNOG00000049913	Rbm12	—	—	molecular function	—
ENSRNOG00000052512	Vps37c	—	—	—	—
ENSRNOG00000053115	Zfp82	—	intracellular	ion binding	nitrogen compound

### Gastrodin intervention inhibited the phosphorylation of PAK2 through regulating PI3K/AKT pathway in diabetic rats

**Figure 3:**
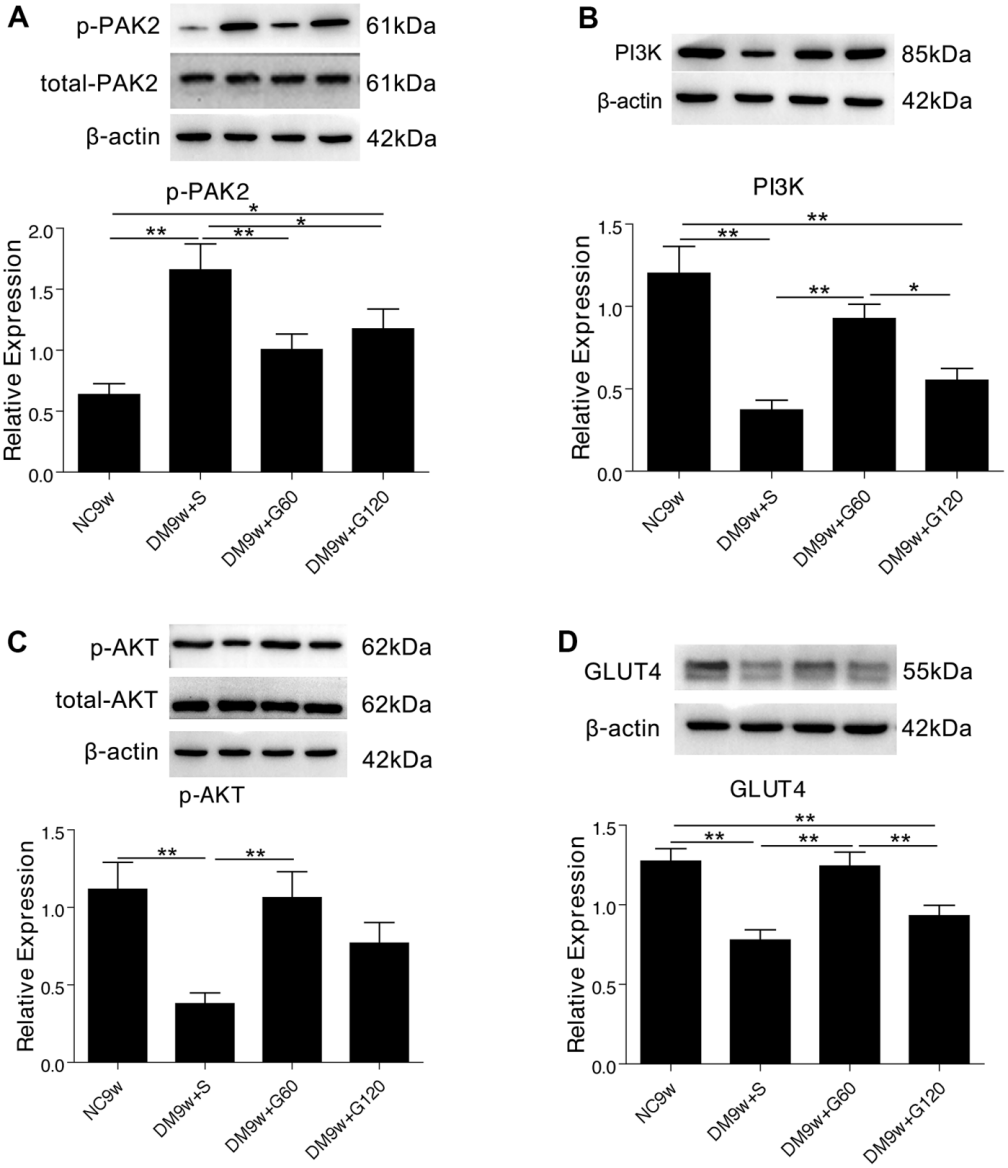
**Gastrodin intervention suppressed diabetes induced PAK2 phosphorylation and activated PI3K/AKT/GLUT4 pathway.** Western blot analysis of p-PAK2 (**A**) PI3K (**B**) p-AKT (**C**) and GLUT4 (**D**) protein expression in the hippocampus of the NC9W, DM9W+S, DM9W+G60 and DM9W+G120 groups. Bar graphs represented optical density of these factors normalized with β-actin, while p-PAK2 and p-AKT were further normalized with total-PAK2 and total-AKT respectively. *p < 0.05 and **p < 0.01.

The expression of p-PAK2 in DM9w+S group was significantly higher than that of NC9w group, while the expression of total-PAK2 showed no significant difference. By contrast, the expression of PI3K, p-AKT and GLUT4 in the hippocampus of DM9w+S group was significantly lower than that of the NC9w group. After 60 mg/kg of Gastrodin intervention, the expression of PI3K, p-AKT, and GLUT4 was significantly increased in diabetic rats, and the expression of p-PAK2 was significantly decreased. However, 120 mg/kg of Gastrodin could only restore the expression of p-PAK2 in diabetic rats (Figure 3).

**Figure 4:**
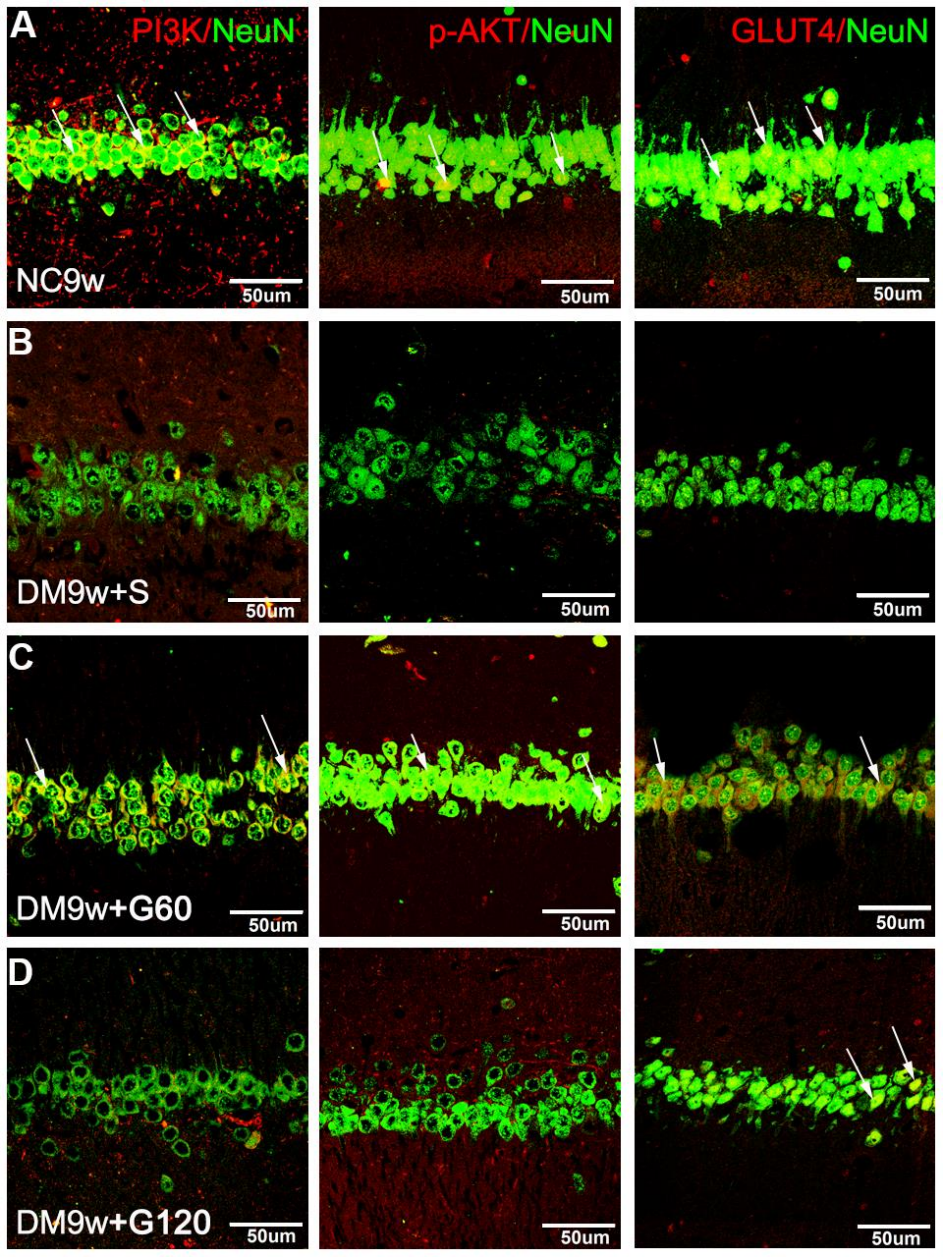
**Gastrodin intervention restored the expression of PI3K/AKT/GLUT4 pathway in the hippocampal neurons of diabetic rats.** Double immunofluorescence staining showed PI3K, p-AKT and GLUT4 positive neurons in the hippocampus of NC9w (**A**) DM9w+S (**B**) DM9w+G60 (**C**) and DM9w+G120 (**D**) groups. Note the diminution of these factors’ immunofluorescence in the neurons of the DM9w+S group as compared with the normal control. However, the immunofluorescence was restored to a level comparable to that of the normal in the DM9w+G60 group. White arrows indicated double positive cells. Bar = 50 μm.

Double immunofluorescence staining showed that PI3K, p-AKT and GLUT4 were localized primarily in the cell body of the hippocampal neurons. The hippocampal neurons emitted intense PI3K immunofluorescence in the NC9w group, which was markedly attenuated in the DM9w+S group. After 60 mg/kg of Gastrodin intervention, the expression of PI3K was restored to a comparable intensity. By contrast, the restoration of PI3K expression was not apparent after 120 mg/kg of Gastrodin intervention. Furthermore, the number of hippocampal neurons was decreased in the DM9w+S group in comparison with the NC9w and DM9w+G60 groups. p-AKT and GLUT4 immunofluorescence in the pyramidal neurons exhibited similar expression changes in the above groups (Figure 4).

### Inhibition of PAK2 improved the survival of hippocampal neurons *in vitro* through PI3K/AKT pathway

**Figure 5:**
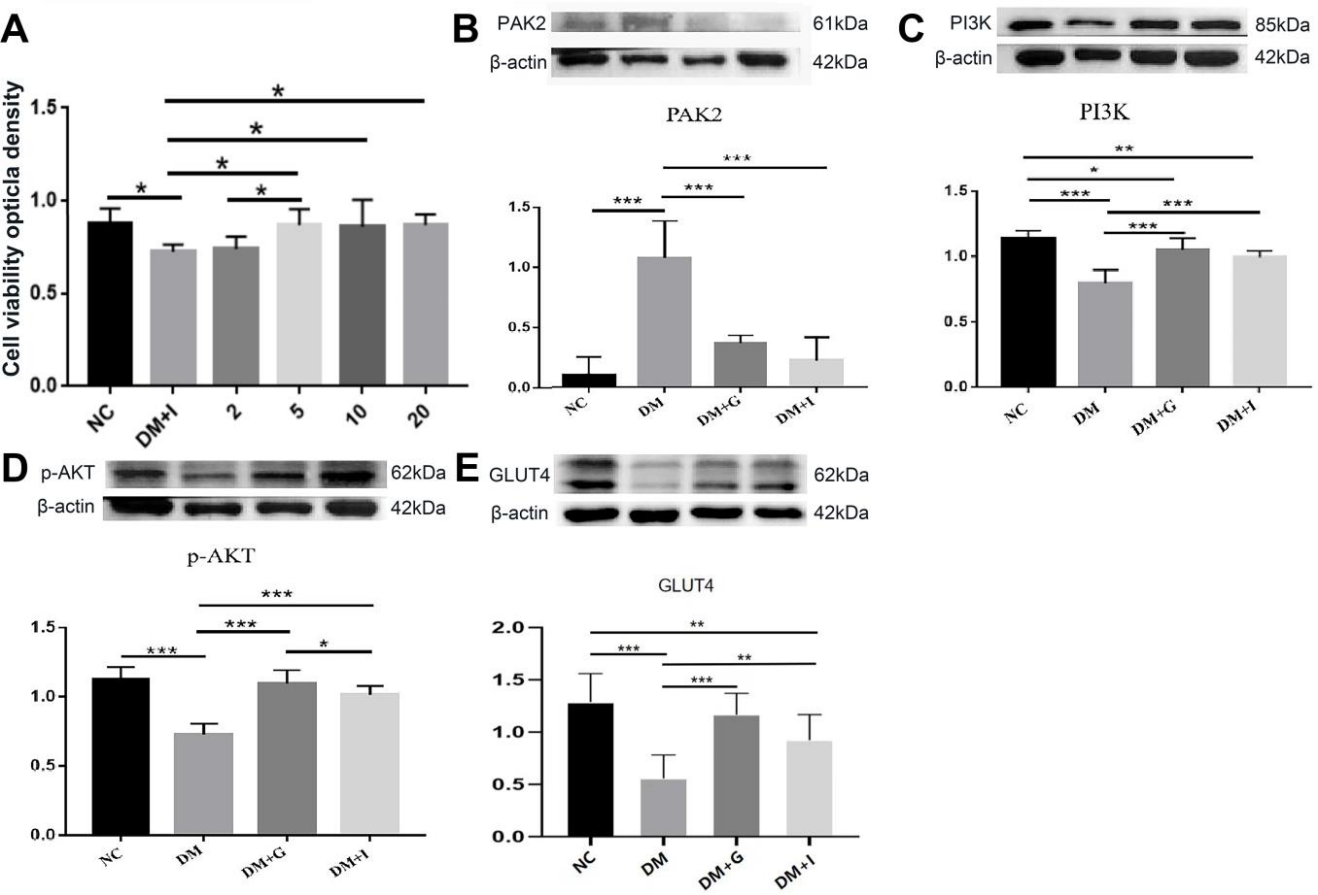
**The effects of Gastrodin intervention and PAK2 inhibition on the PI3K/AKT/GLUT4 pathway in the primary hippocampal neurons during hyperglycemia.** (**A**) CCK-8 analysis of hippocampal neurons exposed to 50 mM glucose in the NC, DM, 2 μM, 5 μM, 10 μM and 20 μM of FRAX597 intervention groups. Western blot analysis of PAK2 (**B**), PI3K (**C**), p-AKT (**D**) and GLUT4 (**E**) protein expression in the primary hippocampal neurons of the NC, DM, DM+G and DM+I groups. Bar graphs represented optical density of these factors normalized with β-actin. *p < 0.05, **p < 0.01 and ***p < 0.001.

To investigate the mechanism of diabetes-induced neuronal death, we examined the effects of PAK2 inhibition on neurotoxicity under high-glucose exposure. CCK-8 analysis of primary hippocampal neurons showed that hyperglycemia caused a significant reduction of cell viability, which could be restored by FRAX597, a small-molecule pyridopyrimidinone, as a potent inhibitor of the group I PAKs. 5, 10 and 20 μM of FRAX597 intervention had a significant effect on the cell viability of primary neurons, while the effect of 2 μM FRAX597 was insignificant (Figure 5A). Since there was no significant difference between the concentration of 5 μM and 20 μM, 5 μM was selected for the subsequent experiments.

**Figure 6:**
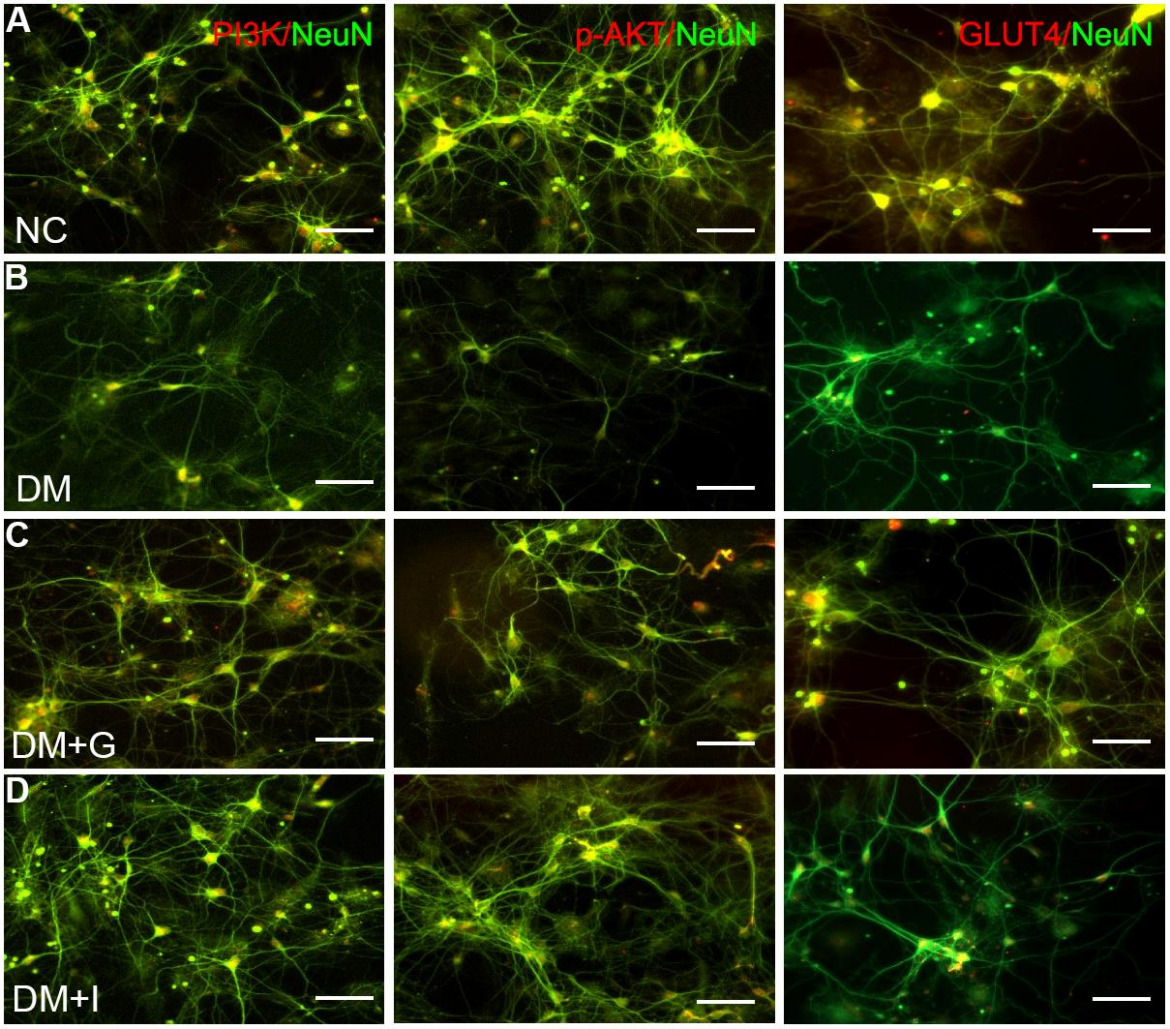
**Gastrodin intervention and PAK2 inhibition restored the expression of PI3K/AKT/GLUT4 pathway in the primary hippocampal neurons exposed to high glucose.** Double immunofluorescence staining of PI3K, p-AKT and GLUT4with NeuN in the primary hippocampal neurons of NC (**A**), DM (**B**), DM+G (**C**) and DM+I (**D**) groups. Bar = 50 μm.

The expression of PAK2, PI3K, p-AKT, and GLUT4 in the primary neurons has shown similar changes compared with the hippocampus. The protein expression of PAK2 was significantly increased after high glucose intervention, while PI3K, p-AKT and GLUT4 was significantly decreased. After both 30 μM of Gastrodin and 5 μM of FRAX597 treatment, the expression of PAK2 was significantly decreased and was significantly and the expression of PI3K, p-AKT and GLUT4 was increased (Figure 5B–5E). Double immunofluorescent staining has shown that PI3K, p-AKT and GLUT4 were distributed mainly in the primary hippocampal neurons. The diminution of the above factors after high glucose exposure could be restored both by Gastrodin and PAK2 inhibitor intervention (Figure 6).

## DISCUSSION

The present study has shown that Gastrodin could inhibit PAK2 phosphorylation and thus ameliorate hippocampal neuronal damage. Our study is the first to screen for the key factors for underlying the therapeutic effects of Gastrodin on diabetes-induced hippocampal injury using RNA-seq. After transcriptome analysis, PAK2 and PI3K/AKT/GLUT4 signaling pathway were selected for further investigation. We compared the expression of PAK2 between normal controls, diabetic rats, and diabetic rats with different doses of Gastrodin intervention. We found that p-PAK2 expression was increased in diabetic rats, while PI3K/AKT/GLUT4 signaling pathway downregulated. PI3K/AKT/GLUT4 signaling pathway was restored after Gastrodin intervention, coupled with decreased p-PAK2 expression. These results indicated that PAK2 activation in diabetes may contribute to decreased GLUT4 expression through PI3K/AKT pathway. Since GLUT4 is related to neuronal glucose uptake, it is suggested that neuronal injury may be caused by decreased neuronal energy. Moreover, therapeutical effects of Gastrodin intervention may be exerted by restoring neuronal glucose uptake through suppressing PAK2 activity.

CID is one of the typical central nervous system complications of DM, but its pathogenesis is far from clear [19]. In the brain, insulin signaling is involved in processes including neurogenesis, cognition, eating behavior, and glucose metabolism [20]. Previous studies have shown that insulin signaling dysfunction is an important mechanism of diabetic complications, including cognitive dysfunction [21]. However, the underlying mechanism of insulin signaling dysfunction is still unknown. The result of our bioinformatic analysis suggested that P21-activated kinase 2 (PAK2) could be an important factor of CID, which may regulate the insulin signaling function and glucose metabolism in the hippocampus.

P21-activated kinase (PAK) is an important component of glucose homeostasis in the muscle, pancreas and liver by mediating insulin signaling [22, 23]. Previous studies have found that PAK2 is involved in neuronal insulin signaling, glucose uptake and insulin resistance [24]. However, its role in neuronal insulin signaling is still unknown [25]. We found that the expression of PAK2 was elevated after diabetes, which suppressed the expression of GLUT4 through PI3K/AKT pathway. Other independent studies have also indicated that PI3K/AKT is involved in the regulation of glucose uptake, which were regulated by PAK2 in the neurons [17]. In Alzheimer’s disease, impaired insulin signaling could inhibit the PI3K/AKT pathway and lead to neurodegeneration by increasing oxidative stress, apoptosis, mitochondrial dysfunction and necrosis [26].

The activity of PI3K/AKT pathway is related to the phosphorylation of AKT. Phosphorylation of AKT, a key downstream protein of PI3K, would be downregulated at high glucose level [24]. This signal transduction dysregulation caused by inhibition of AKT phosphorylation is the key to hyperglycemia induced cognitive deficits [27, 28]. Furthermore, inhibition of AKT and PI3K expression by treatment with AKTi-1/2 and wortmannin reduced insulin regulation of PAK2 and glucose uptake [29].

GLUT4, an insulin-dependent glucose transporter, is involved in the uptake of glucose by neurons [30]. This process is associated with normal cognitive formation [31]. In the neurons, an increase in neurotransmitter activity is also considered as an indirect effect of insulin-dependent glucose uptake, by which insulin regulates cognitive activity [32]. We have found that increased expression of PAK2 in the hippocampus of diabetic rats suppressed GLUT4 expression, which further reduced glucose uptake, supported by a study indicating that overexpression of PAK2 could reduce GLUT4 expression and glucose uptake [33]. Moreover, inhibition PAK2 expression restored the expression of GLUT4 and protect hippocampal neurons.

Finally, we found that Gastrodin intervention had a similar effect on CID as PAK2 inhibitor, suggesting that it could be a potential treatment for CID [34, 35]. Recently, the therapeutic effects of Gastrodin on CNS disorders have been widely investigated due to its capacity to cross the blood-brain barrier. It can be detected in the brain just 5 minutes after intravenous (i.v.) administration at a dose of 50 mg/kg. Despite the relatively low brain-to-blood distribution ratio of Gastrodin, it undergoes metabolism into p-Hydroxybenzyl alcohol, which exhibits similar pharmacological effects [12]. Some clinical trials have found the beneficial outcome of Gastrodin on patients with vascular dementia [12, 36]. Though many studies have found that Gastrodin treated diabetes through PI3K/AKT pathway [37, 38], our study was the first to report that the PAK2 is important for the effect of Gastrodin. Our result also found that 60 mg/kg of Gastrodin intervention had a marginally superior outcome compared to that of 120 mg/kg. However, this difference in latency by Morris water maze test was insignificant. Correspondingly, the protein expression of PI3K and GLUT4 in DM9w+G60 group was higher than the DM9w+G120 group, while that of p-PAK2 and p-AKT showed no significant difference. Therefore, our study showed that the effects of Gastrodin on cognitive function of diabetic rats exhibited similar results between different drug doses.

## CONCLUSIONS

**Figure 7:**
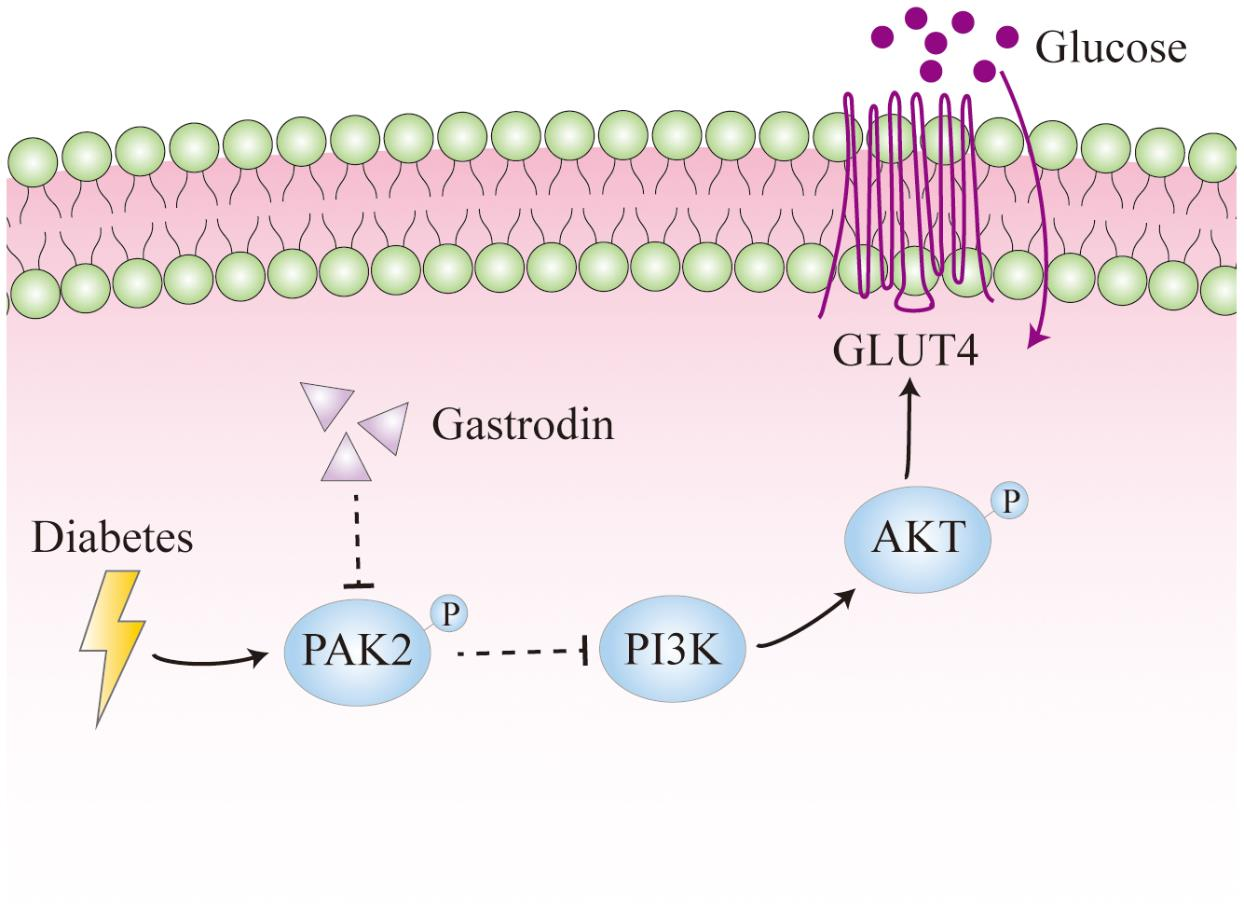
**Diagram illustrating the effects of diabetes on hippocampal neurons.** Diabetes could enhance the phosphorylation of PAK2, which reduces the expression of PI3K and thus the phosphorylation of AKT. It leads to decreased expression of GLUT4, which is important for glucose uptake.

The results obtained from this study have unveiled the detrimental impact of both STZ-induced diabetes and hyperglycemia on hippocampal neurons. These findings suggest a potential underlying mechanism involving elevated phosphorylation of PAK2, leading to reduced expression of PI3K, p-AKT, and GLUT4. Ultimately, this cascade of events may significantly influence glucose utilization, emphasizing the critical role of these molecular pathways in the observed neuronal damage and cognitive dysfunction (Figure 7). Furthermore, Gastrodin could restore the spatial learning ability of the diabetic rats probably through suppressing the phosphorylation of PAK2.

## MATERIALS AND METHODS

### Animal use and care

120 adult male Sprague-Dawley rats (Liaoning Changsheng Biotechnology, Liaoning, China) were housed in our animal facilities in a temperature controlled, pathogen-free room, on a 12:12 light: dark cycle and allowed food and water intake *ad libitum*.

### Diabetes induction and drug administration

The procedure of diabetes induction has been previously described [14]. Briefly, after 2 weeks of adaptation, type 1 diabetes was induced by a single intraperitoneal injection of streptozotocin (65 mg/ kg). Blood glucose levels were assessed by a glucometer and animals were considered as diabetic if the blood glucose levels were higher than 16.7 mmol/l for three consecutive tests.

The rats were randomly divided into 4 groups: (i) NC9w group: normal control rats gavaged with normal saline daily; (ii) DM9w+S group: diabetic rats gavaged with normal saline for 6 weeks at 3 weeks after diabetes induction; (ii) DM9w+G60 group: diabetic rats gavaged with 60 mg/kg/d of Gastrodin for 6 weeks at 3 weeks after diabetes induction; (iv) DM9w+G120 group: diabetic rats gavaged with 120 mg/kg/d of Gastrodin for 6 weeks at 3 weeks after diabetes induction. Gastrodin was acquired from the Kunming Pharmaceutical Corporation.

### Morris water maze test

Morris water maze was performed as previously described [39]. All trials were performed in a quiet room with indirect lighting and the animals would be dried under a heater after each experiment. The apparatus was a circular tank with 190 cm in diameter as a swimming pool and contained water at approximately 22 ± 1° C. Spatial learning was assessed across repeated trials for 7 days. On the first day, the animal was placed into the water for 2 min. On the second day, a circular platform of 15 cm in diameter was positioned 2 cm below the water, and the rats were first put on the platform for 30 s and then guided into the water. If the rats found the platform within 30 s, they would be remaining on it for 30 s. If not, they would be guided to the platform and staying on it for 30 s. Afterwards, the rats were put into the water facing the sidewalls subsequently from one of the four separate quadrants of the pool. If the animal failed to find the platform within 30 s, it would be guided to it and stay there for 30 s. Since the third day, the rats began to be released into the water facing the sidewalls of the pool from one of the quadrants and should find the platform within 120 s. If it succeeds, it would be allowed to stay for 30 s. And if it failed, it would be guided to the platform and stay for 30 s. On the last day, the platform was removed, and the rats were released into the water facing the wall of the pool from the farthest point from the platform. In this test, if the rat recalled the position of the platform, it would swim along a shorter path to the platform on the second trial. The rats not managed to remember the platform’s position in the previous days would not be able to locate it easily. Therefore, we compared the differences among these groups to evaluate their spatial learning ability.

### Hematoxylin and eosin (H&E) staining

To observe the histological changes, the sections were first incubated with hematoxylin (Beyotime Institute of Biotechnology, Shanghai, China) for 5 min and then washed with 1% ethanol hydrochloride for 3 s. After rinsing with water, the sections were stained with eosin. After this, the sections were captured under a light microscope at the magnification ×400 in a blinded manner.

### RNA-sequencing

The samples for RNA-sequencing were collected from the hippocampus of NC9w, DM9+S, DM9w+G60 and DM9w+G120 group. The mRNA libraries were sequenced on the Illumina sequencing platform by Genedenovo Biotechnology Co., Ltd (Guangzhou, China). After the total RNA is extracted from the sample, for eukaryotes, the magnetic beads with Oligo (dT) are used to enrich the mRNA, and the fragmentation buffer is added to the obtained mRNA to make the fragment into a short fragment, and then the fragmented mRNA is used as a template. The first strand of cDNA was synthesized by random hexamers, and the second strand of cDNA was synthesized by adding buffer, dNTPs, RNase H and DNA polymerase I. It was purified by QiaQuick PCR kit and eluted with EB buffer. Add base A, add sequencing linker, and then recover the target size fragment by agarose gel electrophoresis, and carry out PCR amplification to complete the whole library preparation work. The constructed library was sequenced with Illumina HiSeqTM 2500. The SRA data have been uploaded to NCBI (BioProject: PRJNA759189).

### Bioinformatics analysis

The bioinformatics analysis is mainly divided into three modules: First, the TopHat comparison is performed separately for the reads and reference genomes of each sample, and the comparison results of each sample are obtained. Then the cufflinks are used to assemble the transcripts, and the assembly of each sample is obtained. Second, multiple samples were grouped and combined using cuffmerge according to different treatments, the results of different groups were also combined by cuffmerge, and finally the expression levels of the genes in different groups were obtained. Finally, the predicted gene is analyzed by using edgeR for difference analysis, functional annotation of the differential genes and annotation of new genes.

### qPCR

Hippocampal tissues were meticulously isolated and collected with the aim of assessing mRNA levels. RNA extraction was performed using the RNApre pure Tissue Kit reagent (TIANGEN, Beijing, China) in strict accordance with the manufacturer’s recommended protocols. The concentration of extracted RNA was quantified utilizing a spectrophotometer (Bio-Rad, Hercules, CA, USA). After RNA extraction, the reverse transcription reaction was conducted using the RevertAid First Strand cDNA Synthesis Kit (Thermo Fisher Scientific, Waltham, MA, USA), following the provided instructions. The resulting cDNA served as the template for subsequent amplification and quantification, employing the SYBR Green Realtime PCR Master (Solarbio, Beijing, China). The PCR amplification parameters encompassed an initial denaturation step at 95° C for 2 minutes, followed by 45 cycles of denaturation at 95° C for 15 seconds and annealing at 58° C for 15 seconds. To ensure accuracy, each measurement was performed in triplicate. The relative expression of mRNA was determined by normalizing the mRNA levels of PAK2, Mrp1, Sp8, RagA, and RagB to the reference gene GAPDH, employing the 2^(-Δct) method.

### Western blotting analysis

The rats were anesthetized with 10% chloral hydrate administered intraperitoneally. The hippocampal tissues were rapidly dissected and immediately frozen in liquid nitrogen and stored in −80° C. Proteins were extracted from the hippocampal tissues by RIPA buffer (1:1; Beyotime) containing a 1% protease inhibitor cocktail (1:100; Cell Signaling Technology, Danvers, MA, USA) and 1% phosphatase inhibitor cocktails (1:100; Cell Signaling Technology) at 4° C. Homogenates were centrifuged at 12,000 g for 10 min, and the supernatant was collected. Protein concentration was measured using a BCA protein assay kit. The proteins (30 μg) were loaded unto SDS-PAGE gel. The gels were electrophoresed and then transferred to PVDF membranes. After that, the membranes were blocked with a blocking buffer using 5% non-fat milk for 120 min and probed with primary antibodies overnight at 4° C. They were then incubated for 2 h at room temperature with appropriate secondary mouse antibodies (1:1,000; Thermo Fisher Scientific). The following primary antibodies were used for this study: rabbit anti- phosphatidylinositol 3-kinase (PI3K) antibody (1:2000 dilution; Abcam, Cambridge, MA, USA), rabbit anti-AKT antibody (1:2,000 dilution; ABclonal, Woburn, MA, USA), rabbit anti-p-AKT antibody (1:1,000 dilution; CST), rabbit anti-PAK2 antibody (1:2000 dilutions; Abcam), rabbit anti-p-PAK2 antibody (1:1000 dilution; CST), mouse anti-GLUT4 antibody (1:2000 dilution; Santa Cruz, Dallas, TX, USA) and β-tubulin (1:2,000; Santa Cruz). The blots were developed with enhanced chemiluminescence and densitometric analysis of the film was accomplished with ImageJ software (version 1.4.3.67).

### Double immunofluorescence

The hippocampus was dissected, immersed in 4% formaldehyde, dehydrated, cleared with xylene, and embedded in paraffin blocks. Paraffin sections of 4 μm thickness were deparaffinized and hydrated through a series of graded alcohol. The tissues were incubated in citrate buffer for antigen retrieval and the slices were incubated with 5% normal goat serum. The following primary antibodies were used: rabbit anti-PI3K antibody (1:1,00 dilution; Abcam), rabbit anti-p-AKT antibody (1:25 dilution; CST) and mouse anti-GLUT4 antibody (1:20 dilution; Santa Cruz). Primary antibodies were added in a fresh blocking solution and incubated overnight at 4° C. Next, brain sections were incubated with anti-mouse and anti-rabbit Alexa Fluor 488 and 568 secondary antibodies (1:500; A11010, A11001, Invitrogen, Waltham, MA, USA) for 2 h at room temperature and mounted on glass slides. Images were captured on an Olympus FV1000 microscope.

### Primary neuron culture

Primary neurons were prepared as previously described, with minor modifications [40]. Briefly, hippocampus was dissected from the head of postnatal 0 day mice. Hippocampal cells were spread on 6-well plates coated with poly-D-lysine (Sigma-Aldrich, St. Louis, MO, USA) and cultured in DMEM at a density of 25 X 10^5^ cells/well. After 3.5 h of seeding, the medium was changed to Neurobasal medium (Gibco, Carlsbad, NM, USA) supplemented with B-27 (Gibco). Cells were cultured in a humidified incubator at 37° C with 5% CO_2_. Cultures were used for experiments 3 to 7 days after seeding, which were divided into NC, DM (50 mM high glucose for 48 hours), DM+G (50 mM high glucose and 30 μM Gastrodin for 24 hours) and DM+I (50 mM high glucose and 5 μM FRAX597 for 24 hours) groups. FRAX597 (Sigma-Aldrich, St. Louis, MO, USA) is a small molecule selective inhibitor of the p21-activated kinases (PAK).

### CCK8 assay

Cells (4 X 10^4^/well) were seeded in 96-well culture plates and exposed to different conditions as above. Cell viability was then determined by Cell Counting Kit-8 (CCK8) (Beyotime, China) assay. After the exposure, 10 μl of CCK8 solution was added to each well, and the plates were incubated for an additional 2 h at 37° C. Cell viability was measured as the absorbance at 450 nm with a microplate reader (Bio-Rad, USA) and expressed as a percentage of the control level. The mean optical density values from six wells for each treatment were used as the index of cell viability.

### Statistical analysis

All data were expressed as mean ± SD and analyzed by GraphPad Prism8 software. Multiple group comparison was performed with one-way ANOVA analysis to determine significance (Figures 1A–1C, 2D–2H, 3, 5). p < 0.05 was considered statistically significant.

### Data statement

The sequencing data has been deposited into the Sequence Read Archive (accession: PRJNA759189). Other datasets generated during the current study are available from the corresponding author on reasonable request.

### Authors’ approval

All authors have reviewed the contents of the manuscript being submitted, approve of its contents, and validate the accuracy of the data.
